# The risk of preterm birth in women with uterine fibroids: A systematic review and meta-analysis

**DOI:** 10.1371/journal.pone.0269478

**Published:** 2022-06-02

**Authors:** Anadeijda J. E. M. C. Landman, Emma E. Don, Guus Vissers, Hans C. J. Ket, Martijn A. Oudijk, Christianne J. M. de Groot, Judith A. F. Huirne, Marjon A. de Boer

**Affiliations:** 1 Department of Obstetrics and Gynaecology, Amsterdam UMC, Vrije Universiteit Amsterdam, Amsterdam, The Netherlands; 2 Amsterdam Reproduction & Development Research institute, Amsterdam, The Netherlands; 3 Medical Library, Vrije Universiteit Amsterdam, Amsterdam, The Netherlands; 4 Department of Obstetrics and Gynaecology, Amsterdam UMC, University of Amsterdam, Amsterdam Reproduction & Development Research Institute, Amsterdam, The Netherlands; Lausanne University Hospital: Centre Hospitalier Universitaire Vaudois (CH), SWITZERLAND

## Abstract

**Background:**

Fibroids have been identified as a possible risk factor for preterm birth, however, the magnitude of this risk is unclear. Our objective was to determine the risk of total, spontaneous, and medically indicated preterm birth in women with fibroids.

**Methods:**

A literature search was performed on 9 June 2021. We selected studies reporting on preterm birth in women with and without fibroids. Fibroids had to be diagnosed by routine ultrasound before or during pregnancy. Main outcomes were total preterm birth <37, <34, <32, and <28 weeks of gestation, and spontaneous and medically indicated preterm birth. Two authors independently performed study selection, data extraction and quality assessment. We performed quality assessment with the Newcastle-Ottawa scale. Meta-analyses were presented as Odds Ratios (ORs) with 95% Confidence Intervals (95%CIs).

**Main results:**

The search yielded 2078 unique articles of which 11 were included. Meta-analysis for preterm birth <37 weeks of gestation included 256,650 singleton deliveries: 12,309 with fibroids and 244,341 without fibroids. Women with fibroids had a higher rate of preterm birth (11.6% versus 9.0%; OR 1.66, 95%CI 1.29–2.14). Fibroids were also associated with preterm birth <34 (OR 1.88, 95%CI 1.34–2.65), <32 (OR 2.03, 95%CI 1.40–2.95) and <28 (OR 2.24, 95%CI 1.45–3.47) weeks of gestation. Data on type of preterm birth was limited: one study showed a significant association of fibroids with spontaneous preterm birth and another with indicated preterm birth. The main limitations of the included studies were the lack of correction for confounders, the risk of ascertainment bias due to possible underreporting of fibroids, and the substantial heterogeneity between studies.

**Conclusions:**

Our results suggest fibroids are associated with an increased risk of preterm birth, with a stronger risk at earlier gestational ages. We encourage further research to clarify the association between fibroids and preterm birth by systematic myometrial assessment in pregnancy.

**Registration:**

Prospero database [CRD42020186976].

## Introduction

Worldwide 15 million babies are born preterm each year [1]. Preterm birth is the most important cause of perinatal mortality and morbidity [2]. Approximately 65% of preterm births has a spontaneous onset and the remainder is medically indicated for maternal or fetal reasons [3]. The aetiology of preterm birth is multifactorial and complex [4]. Identifying risk factors for preterm birth is important to provide accurate counselling and it might create opportunities for improvement of antenatal care for women who are at risk.

Previous reviews have suggested that women with fibroids have an increased risk of preterm birth [5, 6]. Both associations with spontaneous and medically indicated preterm birth have been described. Several theories on the genesis of spontaneous preterm birth exist, such as a less distensible uterus and increased oxytocin levels, resulting in premature contractions and cervical change [7, 8], Studies have indeed found women with fibroids have a shorter cervix [9, 10]. Fibroids could also disturb uterine contraction patterns [11–14]. Furthermore, the hyperinflammatory state of fibroids could precipitate preterm labour. As described in women with endometriosis, this hyperinflammatory state could impair decidua/trophoblast interaction and uteroplacental development, which, in turn, could increase the risk of hypertensive disorders, placental abruption and fetal growth restriction, for which preterm birth might be medically indicated [15]. Studies have indeed shown an increased risk of these pregnancy complications in women with fibroids [5, 6, 16–19].

To quantify the risk of preterm birth in women with fibroids compared to women without fibroids, we performed a systematic review and meta-analysis. Furthermore, we aimed to differentiate spontaneous from medically indicated preterm birth, and to evaluate how fibroid characteristics (i.e. size, number, localisation) modify the risk of preterm birth.

## Methods

### Protocol and registration

This systematic review was reported according to the PRISMA guidelines for systematics reviews and meta-analyses (**S1 Appendix**) [20]. No funding was received for the conduct of this systematic review and meta-analysis. Ethical approval was not required for secondary use of data in this systematic review and meta-analysis. The protocol was registered in the PROSPERO database in July 2020 (CRD4202018697]. After registration, the following changes were made to the protocol: 1) we only included studies that performed routine ultrasound/imaging for the diagnosis of fibroids to reduce the risk of ascertainment bias; 2) we removed additional outcomes such as preeclampsia as these were not incorporated in the search terms; and 3) we changed the statistical analysis technique from a random-effects model to the generic inverse-variance method to include confounder-adjusted estimates.

### Data sources and search strategies

A systematic literature search was performed by a medical information specialist (JK) and two researchers (GV and AL) on 9 June 2021 in the following databases: PubMed, Embase and Web of Science. The search terms included both keywords as well as free text terms for ‘fibroids’ and ‘preterm birth’, along with their synonyms. The search strategies are presented in **S2 Appendix**. No language, publication date or other restrictions were applied. All duplicates were excluded using EndNote. A cited-reference search was performed to identify potential additional relevant studies.

### Eligibility and study selection

Original studies reporting on the risk of preterm birth in women with and without fibroids were eligible for the present systematic review and meta-analysis. To limit the risk of the opportunistic detection (i.e. incidental identification) of fibroids, the fibroids had to be diagnosed by routine ultrasound or other imaging before or during pregnancy, indicating we excluded studies that, for example, included women that were admitted to the hospital or selectively referred for ultrasound screening. We also excluded studies including women with multiple gestations due to their increased baseline risk of preterm birth, and studies reporting on preterm birth after surgical or non-surgical treatment of fibroids as this treatment may have influenced obstetric outcome. Furthermore, conference abstracts, case reports, case series, reviews and guidelines were excluded. For the purpose of feasibility, we excluded non-English articles. The main outcomes were preterm birth <37, <34, <32 and <28 weeks of gestation. In addition, we aimed to differentiate between the spontaneous onset of preterm birth following either the onset of contractions or rupture of membranes, and medically indicated preterm birth due to maternal or fetal complications. Other relevant outcomes were preterm prelabour rupture of membranes (PPROM) and mid-trimester fetal loss (between 16 and 22 weeks of gestation).

Two authors (GV and AL) independently performed screening of all potential articles using Rayyan [21]. First screening was based on title and abstract. In case of inconsistency between authors, the full article was screened. We screened the full text of the remaining articles based on the aforementioned predefined in- and exclusion criteria for eligibility. Disagreements between the authors were resolved by discussion or consultation of a third author (MdB).

### Data extraction and quality assessment

We used the Newcastle-Ottawa Quality Assessment Scale (NOS) for nonrandomised studies to evaluate the validity of the included studies [22]. We slightly adapted the questions and answers to obtain a better fit for our studies. Each item could be given a maximum of one star. Studies were categorised as good, fair and poor quality according to the Agency for Health Research and Quality standards. Our customised NOS scale and thresholds for quality assessment are demonstrated in **S3 Appendix.** The final judgement of quality was summarised in a risk of bias table. No assessment of certainty was planned in the protocol.

Two authors (GV and AL) independently extracted data from the included articles and performed quality assessment. We collected data on author, year and country of publication, language, publication status, start date and end date of the study, ethical approval, type and source of funding, study design, sample size, population characteristics (e.g. age, ethnicity, parity, obstetrics history, previous cervical cerclage surgery), diagnostics method used for the identification of uterine fibroids, fibroid characteristics (size, location, number, vascularisation, FIGO classification), past treatment of fibroids (surgical or non-surgical), the outcome measures as described previously, and key conclusions. Corresponding authors of the included articles were contacted for further information if relevant outcomes or other data were not presented in the original publication. Discrepancies in data extraction and quality assessment were resolved by discussion and consulting a third author (MdB).

### Statistical analysis

Outcome event rates and crude odds ratios were calculated based on the number of deliveries. If studies included miscarriages in their results, these were excluded from the sample size. If studies were unclear about in- or exclusion of miscarriages in their sample size, we used the reported sample size for our calculations. The meta-analysis included adjusted odds ratios (ORs) of those studies that corrected for confounders and crude ORs of studies that did not. We used a random-effects model with the generic inverse-variance method [23, 24]. As we included both cohort and case-control studies, the results of the meta-analyses were presented as odds ratios and 95% confidence intervals (95% CI). Heterogeneity between studies was evaluated by using *X*^*2*^ and *I*^*2*^ statistics and possible publication bias was assessed with a funnel plot. We performed a pre-planned sensitivity analysis of good-quality studies. In addition, we planned to perform subgroup analyses based on the size, number, location and vascularisation of the fibroids; parity, a history of previous miscarriages or preterm birth, and a history of dilation or curettage. Statistical analyses were performed using Review Manager (RevMan 5.3.5) and R Studio (version 4.0.3: meta, metafor and dmetar packages).

## Results

### Study selection

**Fig 1:**
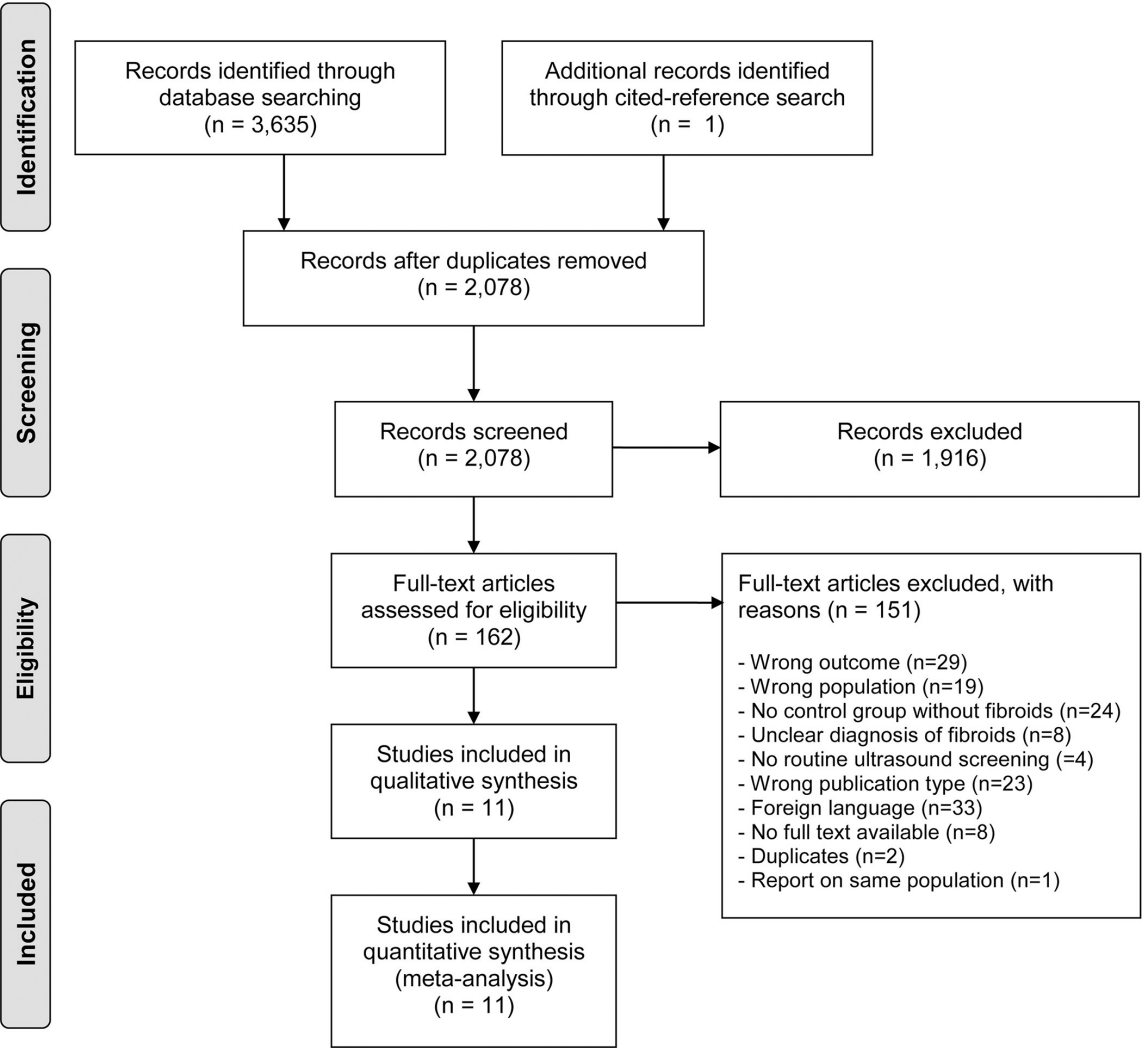
Flow diagram of study selection.

The search provided 2078 unique articles which were assessed for eligibility (Fig 1). One additional relevant article was found through cited-reference search, finally leading to the inclusion of 11 studies and 256,650 deliveries in this systematic review. Two publications reporting on an identical study population and were both used to complement the study data [25, 26]. Characteristics of excluded studies which may appear to meet the inclusion criteria are available in S1 Table [27–38].

### Study and population characteristics

The included studies were conducted between 2009 and 2015. Five of the studies were cohort studies [9, 16, 18, 19, 25, 26, 39] and the other five were case-control studies based on the exposure of fibroids [10, 40–43]. One study was prospective [43]. Study characteristics and sample sizes are demonstrated in Table 1. A total of 256,683 singleton pregnancies with 256,650 deliveries were included in the systematic review: 12,309 in the fibroid group and 244,341 in the control group. The prevalence of fibroids within the cohort studies was 2.9% (6,185/221,813), ranging between 0.8% and 16.8%. All women received routine ultrasound screening (Table 2). Two studies performed screening in the first trimester [16, 39]. Egbe et al. included fibroids >3 cm and Arisoy et al. included intramural fibroids >3 cm [39, 40]. Other studies did not specify a minimum measurement threshold in their exposure definition. Studies did not report whether they used 2D or 3D imaging with or without colour Doppler.

**Table 1:** Study characteristics and sample size.

Study	Year	Country	Study design		Singleton pregnancies	Deliveries	Fibroid group (%)^a^	Control group
Eze et al.	2013	Nigeria	Case control	Prospective	200	174	77	97
			Ratio 1:1					
Zhao et al.	2017	China	Cohort	Retrospective	112,403	Unclear	3,012 (2.7%)	109,391
Stout et al.	2010	USA	Cohort	Retrospective	64,047	Unclear	2,058 (3.2%)	61,989
Chen et al.	2009	Taiwan	Case control	Retrospective	33,762	Unclear	5,627	28,135
			Ratio 1:5					
Girault et al.	2018	France	Cohort	Retrospective	19,719	19,719	154 (0.8%)^b^	19,565
Lai et al.,	2012	USA	Cohort	Retrospective	15,104	15,104	401 (2.7%)	14,703
Qidwai et al.								
Blitz et al.	2016	USA	Cohort	Retrospective	10,314	10,314	522 (5.1%)	9,792
Ciavattini et al.	2015	Italy	Case control	Retrospective	438	438	219	219
			Ratio 1:1					
Arisoy et al.	2016	Turkey	Case control	Retrospective	280	273	106	167
Egbe et al.	2018	Cameroon	Cohort	Retrospective	226	Unclear	38 (16.8%)	188
Shavell et al.	2012	USA	Case control	Retrospective	190	190	95	95
			Ratio 1:1					
**Total**					256,683	256,650	12,309	244,341

^a^ Prevalence of fibroids reported for non-fertility cohort studies only

^b^ This study reported a fibroid prevalence of 1.5% including operated fibroids, which were excluded in the present analysis.

**Table 2:** Methodology of diagnosis of fibroids.

Study	Sample selection	Diagnostic imaging	Timing of ultrasound	Methodology of fibroid measurements	Exposure definition fibroids		
					Fibroid size	Fibroid number	Fibroid location
Eze et al.	Routine ultrasound screening	Ultrasound	Not reported	Size and location	NS	NS	all
				Size: mean of 3 dimensions			
Zhao et al.	All deliveries	Ultrasound	2^nd^ trimester	Not reported	NS	≥ 1	all
			18–22 weeks				
Stout et al.	Routine ultrasound screening	Ultrasound	2^nd^ trimester	Number, size (largest mean diameter), volume (H x W x L), location, relationship to placenta. Description of 6 largest fibroids according to American Institute of Ultrasound in medicine guidelines [44].	NS	≥ 1	all
Chen et al.	Pregnant women who accepted ultrasound screening (national birth registry)	Ultrasound	1st trimester: 73.8%	Diagnosis of fibroids during pregnancy based on ICD-9-CM codes	NS	NS	all
			2nd trimester: 25.2%				
			3d trimester: 1%				
Girault et al.	Routine ultrasound screening	Ultrasound	1^st^ trimester	Number, size, and location	≥1 measuring ≥2 cm, or multiple whatever the size		All
			(11^+0^–13^+6^ weeks)				
Lai et al.,	Routine ultrasound screening	Ultrasound	2^nd^ trimester	Number, size and location	≥ 1 cm	≥ 1	all
Qidwai et al.							
Blitz et al.	Routine ultrasound screening	Ultrasound	2nd trimester	Size (3 dimensions), number and location	NS	≥ 1	all
			17–23 weeks				
Ciavattini et al.	Routine ultrasound screening	Ultrasound	2nd trimester	Size (largest diameter), number (≥2 irrespective of size) and location	NS	NS	all
Arisoy et al.	Routine ultrasound screening	Ultrasound	2nd trimester	Size (3 dimensions) and location	> 3 cm	≥ 1	intramural
			16–24 weeks				
Egbe et al.	All pregnancies	Ultrasound	1^st^ trimester	Size (3 dimensions). Mean of two measurements. Criteria by Muram et al. [45]	> 3 cm	≥ 1	all
Shavell et al.	Routine ultrasound screening	Ultrasound	1^st^ and 2^nd^ trimester	Number, number of fibroids >5cm, diameter largest fibroid, location fibroids >5cm	NS	NS	all
				Volume: H x W x L x π/6			
				Total fibroid volume per patient			

**NS** not specified

Baseline characteristics of the included study populations are described in Table 3. Women with fibroids were generally older and more often black, except in the studies that matched for age and ethnicity. In most studies, women with fibroids had lower parity. Three studies corrected for a previous preterm birth [16, 18, 19]. Blitz et al. excluded women with previous cervical surgery [9]. Eze et al. and Egbe et al. did not adjust for confounders by either statistical analysis or matching of characteristics [39, 43].

**Table 3:** Characteristics of the study population and handling of confounders.

Study	Maternal age (years)			Maternal ethnicity			Parity			Handling of potential confounders
	Fibroid	Control	*P*	Fibroid	Control	*p*	Fibroid	Control	*p*	
Eze et al.	31.6 (±5.5)	29.1 (±5.5)	<0.001	NR	NR	NR	NR	NR	NR	No
Zhao et al.	32.0 (±4.9)	27.9 (±5.2)	<0.001	NR	NR	NR	1.15 (±0.4)	1.21 (±0.5)	<0.01	Adjusted for e.g. maternal age, parity, BMI, HDP, GDM, previous preterm birth.
Stout et al.	35.1 (±4.6)	30.0 (±6.3)	<0.001	Black: 34.5%	Black: 20.3%	<0.001	0.82 (±1.1)	1.06 (±1.2)	<0.01	Adjusted for e.g. maternal age, ethnicity, gravidity, previous preterm birth.
				White: 51.5%	White: 64.0%					
Chen et al.	35–39: 22.6% >39: 4.2%	35–39: 9.3% >39: 1.3%	<0.001	NR	NR	NR	Nulliparous: 55.0%	Nulliparous: 51.7%	<0.001	Adjusted for e.g. maternal age, parity, diabetes, hypertension.
Girault et al.	35.3 (±5.1)	32.0 (±5.4)	<0.001	France: 29.2% Sub-Saharan Africa: 39.6% Other: 31.2%	France: 48.6% Sub-Saharan Africa: 16.1%	<0.001	Nulliparous: 39.0%	Nulliparous: 41.0%	0.62	Adjusted for e.g, maternal age, ethnicity, BMI, parity, previous preterm birth, ART.
					Other: 35.3%					
Lai et al., Qidwai et al.	33.7	28.6	<0.001	Black: 24.2%	Black: 15.7%	<0.001	Nulliparous: 57.4%	Nulliparous 53.7%	<0.001	Adjusted for e.g. maternal age, ethnicity, parity, previous uterine surgery.
				White: 33.1%	White: 38.3%					
				Hispanic: 9.2%	Hispanic: 14.4%					
				Asian: 26.4%	Asian: 25.6%					
Blitz et al.	33.3 (±3.6)	30.9 (±4.5)	<0.001	Black: 26.6%	Black: 6.8%	<0.001	Nulliparous: 67.4%	Nulliparous: 53.9%	<0.001	Excluded women with previous preterm birth.
				White: 43%	White: 66.2%					
				Hispanic: 13.5%	Hispanic: 15.4%					
				Asian 16.9%	Asian: 11.5%					
Ciavattini et al.	34.8 (±4.2)	34.8 (±4.2)	1.0	NR	NR	NR	2.1 (±1)	2 (±1)	0.30	Controls matched for age
Arisoy et al.	34.4 (±4.9)	34.3 (±2.7)	0.83	NR	NR	NR	1.2 (±1.3)	1.3 (±1.0)	0.47	Controls matched for age
Egbe et al.	31.4 (±3.4)	27.4 (±4.2)	<0.001	NR	NR	NR	Nulliparous: 50.0%	Nulliparous: 20.0%	0.02	No
Shavell et al.	Small fibroids: 31.6 (±5.7)	31.9 (±5.6)	0.89	Small fibroids:	Black: 86.3%	0.32	Small fibroids: 2.0 (±1.5)	2.1 (±1.7)	0.14	Controls matched for age.
				Black: 90.5%						
				White: 4.8%						
				Other: 4.8%	White: 10.5%					
					Other: 3.2%					
	Large fibroids: 32.3 (±5.5)			Large fibroids:			Large fibroids: 1.6 (±1.3)			
				Black: 92.5%						
				White: 1.9%						
				Other: 5.7%						

**BMI** Body Mass Index; **HDP** hypertensive disease of pregnancy; **GDM** Gestational Diabetes Mellitus; **NR** not reported

### Risk of bias

According to the Newcastle-Ottawa scale, seven of the included studies were of good quality [10, 16, 18, 19, 25, 26, 41, 42], and four of poor quality [9, 39, 40, 43]. See S1 Fig for the complete risk of bias assessment. The most important reasons for a fair or poor quality rating of studies were: not controlling or matching for important confounders such as maternal age and ethnicity, and high or unknown rates of loss to follow-up. The funnel plot of included studies has a fairly symmetrical pattern, indicating there is no clear evidence of publication bias (S2 Fig).

### Preterm birth

**Fig 2:**
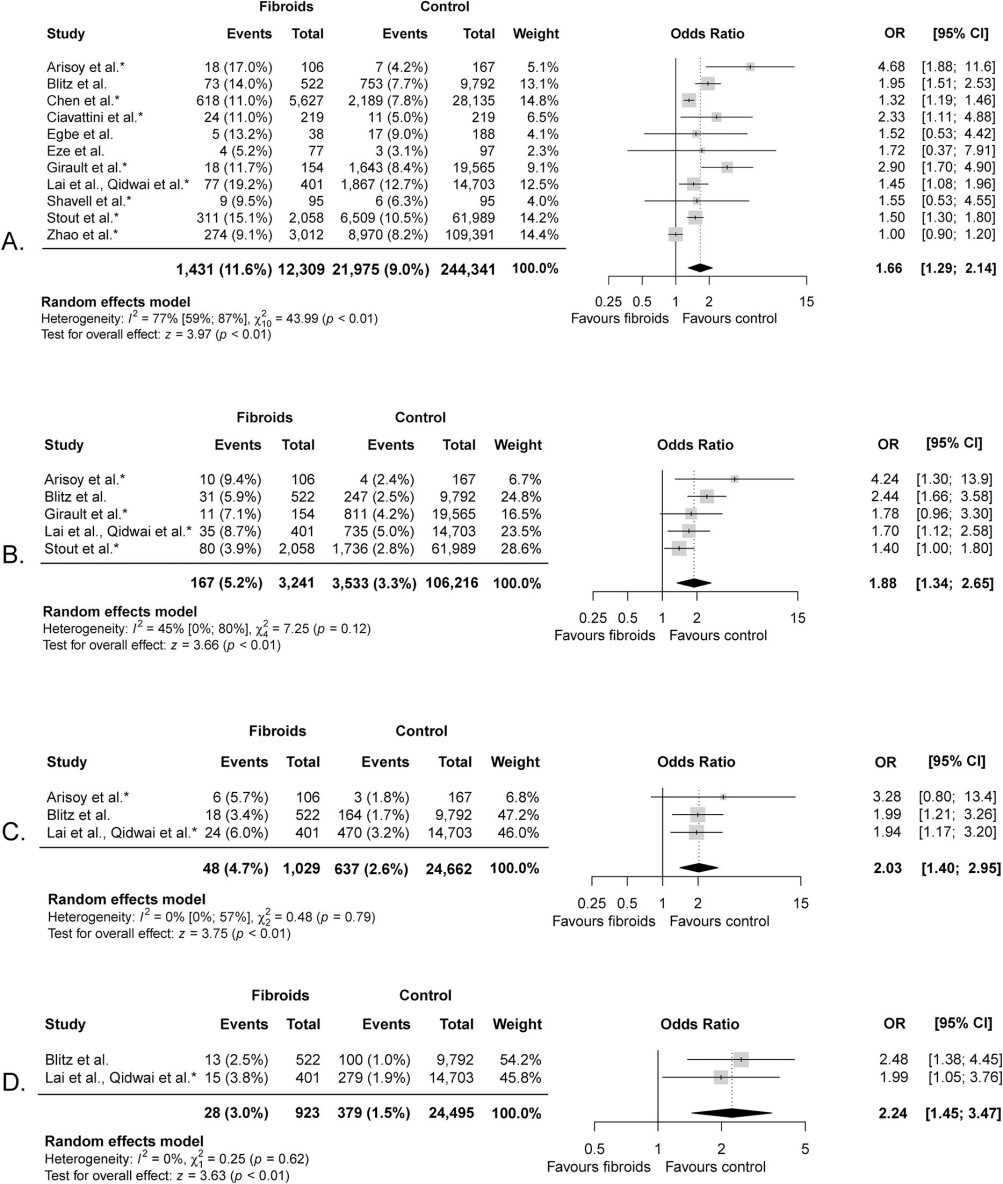
Meta-analysis of preterm birth <37 (A.), <34 (B.), <32 (C.) and <28 (D.) weeks of gestation in women with fibroids. * indicates studies that corrected for potential confounders.

Preterm birth was defined as birth <37 weeks of gestation by all studies and several studies made subgroups at other gestational age cut-offs. The lower limit of the gestational age cut-off mostly varied between 20–24 weeks of gestation. Zhao et al. and Egbe et al. used a cut-off at 28 weeks of gestation (S2 Table) [19, 39]. Pooled preterm birth rates <37 weeks of gestation were 11.6% in the fibroid group and 9.0% in the control group (Fig 2A). The meta-analysis of 256,650 women showed women with fibroids had an increased risk of preterm birth <37 weeks compared to women without fibroids (OR 1.66, 95% CI 1.29–2.14). Heterogeneity was considerable (X^2^ = 43.99, p<0.01; I^2^ = 77%). Meta-analyses also showed a significant association of fibroids with preterm birth <34, <32, and <28 weeks of gestation and the strength of this association increased with preterm birth at earlier gestational ages (Fig 2B–2D). Meta-analyses for preterm birth <34 weeks of gestation included five studies with 109,457 women (5.2% versus 3.3%; OR 1.88, 95% CI 1.34–2.65), <32 weeks of gestation included three studies with 25,691 women (4.7% versus 2.6%; OR 2.03, 95% CI 1.40–2.95) and <28 weeks gestation included two studies with 25,418 women (3.0% versus 1.5%; OR 2.24, 95% CI 1.45–3.47). We did not identify any studies who reported on mid-trimester fetal losses between 16 and 22 gestational weeks.

Only Girault et al. differentiated between spontaneous and medically indicated preterm birth <37 weeks of gestation. They found that women with fibroids are at increased risk of medically indicated preterm birth compared to women without fibroids (RR 1.99, 95% CI 1.18–3.36). The medical indications leading to these preterm birth were not reported. There was no difference in spontaneous preterm birth between the fibroid group (5/154 = 3.2%) compared to the control group (820/19,565 = 4.2%); RR 0.77 (95% CI 0.33–1.84) [16]. Conversely, Ciavattini et al. did show an increased risk of spontaneous preterm birth in women with fibroids (OR 2.33, 95% CI 1.11–4.88) [42].

### PPROM

**Fig 3:**
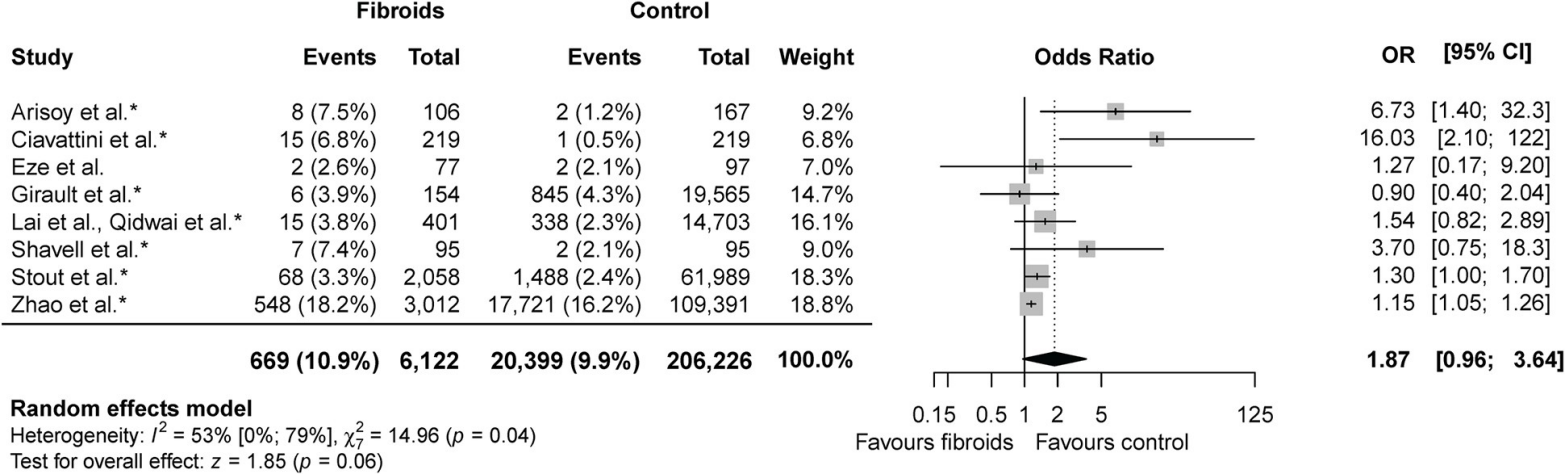
Meta-analysis PPROM. * indicates studies that corrected for potential confounders.

Eight studies reported on PPROM, including 212,348 women: 6,122 in the fibroid group and 206,226 in the control group [10, 16, 18, 19, 25, 26, 41, 42]. Meta-analysis showed no difference in PPROM rate between the fibroid and the control group (10.9% versus 9.9%; OR 1.87, 95% CI 0.96–3.64) (Fig 3). Statistical heterogeneity was substantial (X^2^ = 14.96, p = 0.04; I^2^ = 53%). An overview of the crude and adjusted effect sizes per study and outcome can be found in S3 Table.

### Sensitivity analyses

Good quality studies were included in sensitivity analyses (S3 Fig). All these studies adjusted for potential confounders. The meta-analyses showed a smaller, yet still significant, association between fibroids and preterm birth <37 (11.5% versus 9.1%; OR 1.50, 95% CI 1.15–1.95) and <34 (4.7% versus 3.2%; OR 1.50, 95% CI 1.16–1.94) weeks of gestation. A meta-analysis of preterm birth <32 and <28 weeks of gestation could not be performed as there was one good quality study assessing these outcomes. There was no significant association between fibroids and PPROM in a sensitivity analysis (11.3% versus 10.5%; OR 1.97, 95% CI 0.84–4.61). Girault et al. was rated as good quality but was not included in sensitivity analyses for preterm birth <34 weeks of gestation and PPROM [16]. They provided additional preterm birth rates in their unoperated fibroid group upon our request. As we did not have the adjusted ORs for these particular outcomes, this lead to the deduction of points in quality in the comparability section with regards to these outcomes.

### Subgroups based on fibroid characteristics

The results of the subgroup analyses based on fibroid characteristics are shown in S4 Fig. Subgroup analysis including studies comparing women with fibroids >3 cm and >5 cm to women without fibroids showed that, overall, there was a significant association between fibroids and preterm birth <37 weeks of gestation (n = 177,521; OR 2.03, 95% CI 1.26–3.27). Heterogeneity was substantial (X^2^ = 13.29, p = 0.02; I^2^ = 62%). Meta-analyses did not show differences in preterm birth <37 weeks of gestation when comparing large (>5 cm) to small (≤5 cm) fibroids (OR 1.38, 95% CI 0.78–2.46), nor when comparing multiple to single fibroids (OR 1.89, 95% CI 0.79–4.53). No subgroup analyses could be performed on fibroid location (submucosal, intramural, subserosal), vascularisation, FIGO classification or other ultrasound features. Neither could we perform subgroup analyses based on parity, a history of previous mid-trimester fetal losses or preterm birth, or a history of dilation and curettage.

## Discussion

The present meta-analysis, including 11 studies and 256,650 deliveries, suggests uterine fibroids are associated with preterm birth. This association was present for preterm birth <37 weeks of gestation and became stronger with preterm birth at earlier gestational ages <34, <32 and <28 weeks. Moreover, the association with preterm birth <37 and <34 weeks of gestation was also significant in sensitivity analyses including good-quality studies. No association was found between PPROM and the presence of fibroids. No distinction could be made between spontaneous and medically indicated preterm birth in meta-analyses. This distinction is crucial for the interpretation the study results due the disparate underlying aetiologies of these types of preterm birth. Only two included studies made this distinction: one included study showed an association between spontaneous preterm birth and the other showed an association with medically indicated preterm birth. No conclusions can be drawn from these limited data.

There is insufficient evidence to conclude whether the size or number of fibroids modifies the risk of preterm birth or not. Other potentially relevant fibroid characteristics have not been reported in the included studies. Fibroid activity, for instance, may be important in the genesis of preterm birth. Fibroid activity could be determined by assessing vascularisation, fibroid growth, signs of degeneration, signs of calcification or other MUSA criteria [46, 47].

Two past reviews also found an association between fibroids and preterm birth. Klatsky et al. found a preterm birth rate of 16.0% in the fibroid group and 10.8% in the control group (OR 1.5, 95% CI 1.3–1.7), and Perez-Roncero et al. of 11.7% versus 9.0% (OR 1.43; 95% CI 1.27–1.60) [5, 6]. These reviews also included studies that did not perform routine ultrasound screening during pregnancy, which are at risk of ascertainment bias, and they did not quantitatively address possible bias resulting from important confounders in their meta-analyses. Furthermore, the studies did not aim to distinguish between spontaneous and medically indicated preterm birth [5, 6].

We did not identify any studies that reported on mid-trimester fetal loss between 16 and 22 weeks of gestation. These mid-trimester fetal losses are often included in the group of miscarriages defined as loss <20–24 weeks of gestation. A recent systematic review and meta-analysis concluded that the risk of miscarriage, including mid-trimester fetal losses, was not increased in women with fibroids in a general obstetric population [48]. However, we presume that first-trimester fetal losses and mid-trimester losses have different aetiologies and, therefore, we propose future studies to make a distinction between these entities [49].

We performed an in-depth evaluation of preterm birth in women with fibroids, and a meta-analysis including confounder-adjusted effect sizes. We also performed sensitivity analyses including good-quality studies. Nevertheless, the results of our study should be interpreted in light of the following considerations. Not all studies controlled for or matched for all relevant confounders (e.g. cervical surgery, uterine malformations, socioeconomic status), and there still might be residual confounding present.

We minimised the risk of ascertainment bias by excluding studies with women who were selectively referred for ultrasound screening. However, even when routine prenatal ultrasound screening is performed, there is a risk of underreporting of fibroids in the absence of a prospective protocol for myometrial assessment because the primary focus lies on the fetus rather than the uterine wall. Moreover, ultrasound screening in the second and third trimester also leads to underreporting, as not all fibroids, especially those located in the posterior uterine wall, can be visualised due to the size of the fetus. It seems impossible to quantify the frequency of undiagnosed fibroids in these studies. As the risk of the opportunistic recording of fibroids is likely smaller with larger fibroids, we performed a subgroup analysis including studies that compared fibroids >3 cm and >5 cm to women without fibroids. In this analysis, there was still a significant association present between fibroids and preterm birth. This association was stronger for fibroids >3 cm than for fibroids >5 cm, but uncertainty is large due to the wide confidence interval in the subgroup of fibroids >3 cm. This is probably a consequence of a small number of studies, including one study with an outlying effect size.

Finally, there was substantial heterogeneity between studies, which might have been caused by variations in study populations and methodology. There was a wide range in the prevalence of fibroids between studies. This is probably a reflection of differences in ethnicity, as the lowest prevalence of fibroids was found in a French study and the higher prevalence in a Cameroonian study. However, selection bias resulting from opportunistic reporting could also play a role. Furthermore, variations in exposure definitions of fibroids and lower gestational age cut-offs may have caused heterogeneity by differential misclassification.

Based on our findings, systematic myometrial assessment before or during the first trimester of pregnancy is warranted. Future studies should have a protocol for prospective fibroid assessment on ultrasound including evaluation of fibroid size, number, FIGO classification, location (corporeal or cervical) growth, and ultrasound features such as vascularisation, degeneration, and calcification [46, 47]. Registration of these characteristics may provide insight into the association with preterm birth and the underlying pathophysiologic mechanism(s). This, in turn, could give more insight as to which screening- and preventative strategies could be helpful to improve antenatal care for women with fibroids.

## Conclusions

This systematic review and meta-analysis suggests fibroids are associated with total preterm birth and this association is stronger with earlier gestational age of the preterm birth. However, the considerable amount of heterogeneity between studies may indicate biased results. Considering the magnitude of the disease burden of preterm birth, as well as the biological plausibility of an association between preterm birth and fibroids, we encourage further research to clarify this association through prospective and systematic myometrial assessment in early pregnancy. Finally, it is important that these studies distinguish between spontaneous and medically indicated preterm birth.

## Supporting information

S1 Appendix PRISMA guidelines. (PDF)

S2 Appendix Search strategy. (PDF)

S3 Appendix Modified Newcastle Ottawa quality assessment scale and AHRQ standards. (PDF)

S1 Table Characteristics of excluded studies. (PDF)

S2 Table Definitions of preterm birth per study. (PDF)

S3 Table Crude and adjusted effect sizes of preterm birth and PPROM. (PDF)

S1 Fig Risk of bias assessment and overall quality of studies. (TIF)

S2 Fig Funnel plot of preterm birth <37 weeks of gestation. (TIF)

S3 Fig Sensitivity analyses of good quality studies for preterm birth (A) <37 and (B) <34 gestational weeks and (C) PPROM. * indicates studies that corrected for potential confounders.
(TIF)

S4 Fig Meta-analysis of (A) fibroids >3 and >5 cm compared to women without fibroids; (B) large versus small fibroids; and (C) multiple versus single fibroids. * indicates studies that corrected for potential confounders.
(TIF)
