# Supplementary material for: The risk of preterm birth in women with uterine fibroids: A systematic review and meta-analysis
Source: PLoS One. 2022 Jun 2;17(6):e0269478. doi: 10.1371/journal.pone.0269478 (PMC9162311; doi:10.1371/journal.pone.0269478)
Supplement: S2 Appendix — (PDF) [file pone.0269478.s002.pdf]

S2 Appendix – Search strategy

PubMed 9 jun 2021 (1106)

Search	Query	Items found
#3	#1 AND #2	1106
#2	"Obstetric Labor, Premature"[Mesh] OR "Infant, Premature"[Mesh] OR "Fetal Membranes, Premature Rupture"[Mesh:NoExp] OR "Abortion, Spontaneous"[Mesh] OR prematur*[tiab] OR preterm*[tiab] OR "pre matur*"[tiab] OR preterm*[tiab] OR "pre term*"[tiab] OR ptb[tiab] OR ptbs[tiab] OR prom[tiab] OR proms[tiab] OR pprom[tiab] OR pproms[tiab] OR abortion*[tiab] OR miscarr*[tiab] OR "pregnancy loss*"[tiab] OR "loss of pregnanc*"[tiab] OR "embryo loss*"[tiab] OR "embryo death*"[tiab] OR "embryo resorption*"[tiab] OR "embryo disintegration*"[tiab] OR "disintegration of embryo*"[tiab]	331453
#1	"Myoma"[Mesh:NoExp] OR "Leiomyoma"[Mesh] OR myoma*[tiab] OR leiomyoma*[tiab] OR adenomyoma*[tiab] OR fibroid*[tiab] OR fibroma*[tiab] OR fibromyoma*[tiab] OR angiomyoma*[tiab] OR angioleiomyoma*[tiab]	45251

Embase.com 9 jun 2021 (1750)

Search	Query	Items found
#4	#3 NOT 'conference abstract'/it	1750
#3	#1 AND #2	2277
#2	'immature and premature labor'/exp OR 'premature fetus membrane rupture'/exp OR 'spontaneous abortion'/exp OR 'second trimester abortion'/exp OR 'embryo death'/exp OR 'pregnancy loss'/exp OR 'embryo resorption'/exp OR prematur*:ti,ab,kw OR 'pre matur*':ti,ab,kw OR preterm*:ti,ab,kw OR 'pre term*':ti,ab,kw OR ptb:ti,ab,kw OR ptbs:ti,ab,kw OR prom:ti,ab,kw OR proms:ti,ab,kw OR pprom:ti,ab,kw OR pproms:ti,ab,kw OR abortion*:ti,ab,kw OR miscarr*:ti,ab,kw OR 'pregnancy loss*':ti,ab,kw OR 'loss of pregnanc*':ti,ab,kw OR 'embryo loss*':ti,ab,kw OR 'embryo death*':ti,ab,kw OR 'embryo resorption*':ti,ab,kw OR 'embryo disintegration*':ti,ab,kw OR 'disintegration of embryo*':ti,ab,kw	456063
#1	'myoma'/exp OR 'adenomyoma'/exp OR 'fibromatosis'/de OR 'fibroma'/de OR myoma*:ti,ab,kw OR leiomyoma*:ti,ab,kw OR adenomyoma*:ti,ab,kw OR fibroid*:ti,ab,kw OR fibroma*:ti,ab,kw OR fibromyoma*:ti,ab,kw OR angiomyoma*:ti,ab,kw OR angioleiomyoma*:ti,ab,kw	74776

Web of Science Core Collection 9 jun 2021 (779)

Search	Query	Items found
#3	#1 AND #2	779
#2	TS=("prematur*" OR "preterm*" OR "pre matur*" OR "preterm*" OR "pre term*" OR "ptb" OR "ptbs" OR "prom" OR "proms" OR "pprom" OR "pproms" OR "abortion*" OR "miscarr*" OR "pregnancy loss*" OR "loss of pregnanc*" OR "embryo loss*" OR "embryo death*" OR "embryo resorption*" OR "embryo disintegration*" OR "disintegration of embryo*")	324813
#1	TS=("myoma*" OR "leiomyoma*" OR "adenomyoma*" OR "fibroid*" OR "fibroma*" OR "fibromyoma*" OR "angiomyoma*" OR "angioleiomyoma*")	35394
