# Supplementary material for: The risk of preterm birth in women with uterine fibroids: A systematic review and meta-analysis
Source: PLoS One. 2022 Jun 2;17(6):e0269478. doi: 10.1371/journal.pone.0269478 (PMC9162311; doi:10.1371/journal.pone.0269478)
Supplement: S3 Appendix — (PDF) [file pone.0269478.s003.pdf]

S3 Appendix – Modified Newcastle Ottawa quality assessment scale and AHRQ standards

COHORT STUDIES

Selection

1. Representativeness of the fibroid group for the general for the pregnant population
    - A. truly representative *
    - B. somewhat representative * (e.g. tertiary care hospital)
    - C. selected group (e.g. women receiving ART, selection based on hospital admission)
    - D. no description of the derivation of the cohort

2. Selection of non-exposed cohort (same source population)
    - A. drawn from the same community as the exposed cohort *
    - B. drawn from a different source
    - C. no description of the derivation of the non-exposed cohort

3. Diagnosis of fibroids with imaging
    - A. ultrasound or other imaging studies; obtained from secured records *
    - B. structured interview *
    - C. written self-report
    - D. no description

4. Definition of controls - exclusion of fibroids in control group
    - A. Absence of fibroids confirmed with imaging *
    - B. no

Comparability

1. Comparability of cases and controls on the basis of the design or analysis
    Study controls for ethnicity *
    Study controls for maternal age *

Outcome

1. Assessment of outcome preterm birth
    - A. independent blind assessment *
    - B. record linkage *
    - C. self-report
    - D. no description

2. Was follow-up long enough for outcomes to occur (until delivery)
    - A. yes *
    - B. no/not described

3. Adequacy of follow-up of cohorts
    - A. complete follow up until delivery - all subjects accounted for *
    - B. subjects lost to follow-up unlikely to introduce bias - small number lost: <10%
    - C. follow-up rate <90% and no description of those lost
    - D. no statement or insufficient information on missing data (e.g. exclusion of patients delivering at other center)

CASE CONTROL STUDIES

Selection

1. Is the case definition of fibroids adequate?
   - A. routine ultrasound examination during pregnancy *
   - B. record linkage (ICD codes), self-reports
   - C. no description

2. Representativeness of the cases
   - A. consecutive or obviously representative cases *
   - B. potential for selection biases or not stated

3. Selection of controls
   - A. controls from same community (same source population) *
   - B. hospital controls/different source
   - C. no description

4. Definition of controls - exclusion of fibroids in control group;
   - A. Absence of fibroids confirmed with imaging *
   - B. No description/no imaging

Comparability

1. Comparability of cases and controls on the basis of the design or analysis
   Study matches or controls for ethnicity
   Study matches or controls for maternal age

Outcome

1. Ascertainment of outcome preterm birth
   - A. secure record (hospital records, registry data) *
   - B. structured interview where blind to case/control status *
   - C. interview not blinded to case/control status
   - D. written self-report or medical record only
   - E. no description

2. Same method of ascertainment of the outcome preterm birth for cases & controls
   - A. yes *
   - B. no

3. Non-response rate/loss to follow-up/missing outcome data
   - A. same rate for both groups *
   - B. non respondents described
   - C. rate different and no designation

Thresholds for converting the Newcastle-Ottawa scales to AHRQ standards (good, fair, and poor):
Good quality: 3 or 4 stars in selection domain AND 1 or 2 stars in comparability domain AND 2 or 3 stars in outcome/exposure domain
Fair quality: 2 stars in selection domain AND 1 or 2 stars in comparability domain AND 2 or 3 stars in outcome/exposure domain
Poor quality: 0 or 1 star in selection domain OR 0 stars in comparability domain OR 0 or 1 stars in outcome/exposure domain
