# Supplementary material for: The risk of preterm birth in women with uterine fibroids: A systematic review and meta-analysis
Source: PLoS One. 2022 Jun 2;17(6):e0269478. doi: 10.1371/journal.pone.0269478 (PMC9162311; doi:10.1371/journal.pone.0269478)
Supplement: S1 Table — (PDF) [file pone.0269478.s004.pdf]

S1 Table Characteristics of excluded studies

Study	Year	Country	Study design		Reason for exclusion
Conti et al.[1]	2013	Italy	Cohort	Retrospective	Unclear diagnosis of fibroids.
Davis et al.[2]	1990	USA	Case control Ratio 1:1	Prospective	Fibroid diagnosis not by routine ultrasound. "The history of uterine leiomyomas was based on either a clinical assessment by pelvic examination or previous intraoperative evaluation."
Dogan et al.[3]	2016	Turkey	Case control Ratio 1:1	Retrospective	Fibroid diagnosis not by routine ultrasound. Inclusions were consecutive pregnant women who were admitted to the tertiary care hospital.
Exacoustos et al.[4]	1993	Italy	Cohort	Retrospective	Fibroid diagnosis not by routine ultrasound. Women with an admission between 6 and 42 weeks of gestation were included.
Harlev et al.[5]	2019	Israel	Cohort	Retrospective	Unclear diagnosis of fibroids. "Exposure was defined as known maternal fibroid uterus".
Karlsen et al.[6]	2020	Denmark	Cohort	Retrospective	Unclear fibroid diagnosis. The exposure definition of fibroids was based on International Classification of Diseases (ICD10).
Lee et al.[7]	2020	Republic of Korea	Cohort	Retrospective	Unclear fibroid diagnosis. The exposure definition of fibroids was based on International Classification of Diseases (ICD10).
Majeed et al.[8]	2011	Pakistan	Case control Ratio 1:1	Unclear	Unclear diagnosis of fibroids. "In the study group, all the patients having singleton pregnancy associated with fibroid uterus or directly presented to the labour ward (..) were included."
Murata et al.[9]	2021	Japan	Cohort	Retrospective	Unclear exposure definition. "Uterine myomas were diagnosed before pregnancy or at the first trimester through ultrasonography." However, it is also reported that: "Women with uterine myomas were identified based on a self-reported questionnaire".
Oliveira et al.[10]	2005	Brazil	Case control Ratio 1:1	Retrospective	Fibroid diagnosis not by routine ultrasound during pregnancy. Selected population from a IVF-ET centre.
Oztekin et al.[11]	2012	Turkey	Cohort	Retrospective	Unclear diagnosis of fibroids.
Sheiner et al.[12]	2004	Israel	Cohort	Retrospective	Unclear diagnosis of fibroids.

References

1. Conti N, Tosti C, Pinzauti S, Tomaiuolo T, Cevenini G, Severi FM, et al. Uterine fibroids affect pregnancy outcome in women over 30 years old: role of other risk factors. J Matern Fetal Neonatal Med. 2013;26(6): 584-587.
2. Davis JL, Ray-Mazumder S, Hobel CJ, Baley K, Sassoon D. Uterine leiomyomas in pregnancy: a prospective study. Obstet Gynecol. 1990;75(1): 41-44.
3. Doğan S, Özyüncü Ö, Atak Z. Fibroids During Pregnancy: Effects on Pregnancy and Neonatal Outcomes. The Journal of reproductive medicine. 2016;61(1-2): 52-57.
4. Exacoustòs C, Rosati P. Ultrasound diagnosis of uterine myomas and complications in pregnancy. Obstet Gynecol. 1993;82(1): 97-101.
5. Harlev A, Wainstock T, Walfisch A, Landau D, Sheiner E. Perinatal outcome and long-term pediatric morbidity of pregnancies with a fibroid uterus. Early Hum Dev. 2019;129: 33-37.
6. Karlsen K, Schiøler Kesmodel U, Mogensen O, Humaidan P, Ravn P. Relationship between a uterine fibroid diagnosis and the risk of adverse obstetrical outcomes: a cohort study. BMJ open. 2020;10(2): e032104.
7. Lee SJ, Ko HS, Na S, Bae JY, Seong WJ, Kim JW, et al. Nationwide population-based cohort study of adverse obstetric outcomes in pregnancies with myoma or following myomectomy: retrospective cohort study. BMC Pregnancy Childbirth. 2020;20(1): 716.
8. Majeed T, Waheed F, Sattar Y, Mobusher I, Saba K. Impact of uterine fibroids on the obstetric performance of the women; Complications and pregnancy outcome. Pakistan Journal of Medical and Health Sciences. 2011;5: 274-277.
9. Murata T, Kyozuka H, Endo Y, Fukuda T, Yasuda S, Yamaguchi A, et al. Preterm Deliveries in Women with Uterine Myomas: The Japan Environment and Children's Study. Int J Environ Res Public Health. 2021;18(5).
10. Oliveira FG, Abdelmassih VG, Diamond MP, Dozortsev D, Melo NR, Abdelmassih R. Impact of subserosal and intramural uterine fibroids that do not distort the endometrial cavity on the outcome of in vitro fertilization-intracytoplasmic sperm injection. Fertility and sterility. 2004;81(3): 582-587.
11. Oztekin D, Sofuoglu K, Caliskan E, Kars B, Cetinkaya T, Tug N. Effects of leiomyomas less than 5 cm in diameter on the outcomes of assisted reproductive technology. Ginecoro. 2012;8: 128-130.
12. Sheiner E, Bashiri A, Levy A, Hershkovitz R, Katz M, Mazor M. Obstetric characteristics and perinatal outcome of pregnancies with uterine leiomyomas. The Journal of reproductive medicine. 2004;49(3): 182-186.
