# Supplementary material for: The risk of preterm birth in women with uterine fibroids: A systematic review and meta-analysis
Source: PLoS One. 2022 Jun 2;17(6):e0269478. doi: 10.1371/journal.pone.0269478 (PMC9162311; doi:10.1371/journal.pone.0269478)
Supplement: S2 Table — (PDF) [file pone.0269478.s005.pdf]

S2 Table Definitions of preterm birth per study

Study	Type of preterm birth	Lower gestational age cut-off
Eze et al.	Total	Not reported
Zhao et al.	Total	28
Stout et al.	Total	Not reported
Chen et al.	Total	Not reported
Girault et al.	Total, spontaneous and indicated	22
Lai et al., Qidwai et al.	Total	24
Blitz et al.	Total	20
Ciavattini et al.	Spontaneous	24
Arisoy et al.	Total	20
Egbe et al.	Total	28
Shavell et al.	Total	20
