# Supplementary material for: The risk of preterm birth in women with uterine fibroids: A systematic review and meta-analysis
Source: PLoS One. 2022 Jun 2;17(6):e0269478. doi: 10.1371/journal.pone.0269478 (PMC9162311; doi:10.1371/journal.pone.0269478)
Supplement: S3 Table — (PDF) [file pone.0269478.s006.pdf]

S3 Table Crude and adjusted effect sizes of preterm birth and PPROM

Study	Preterm birth <37 weeks		Preterm birth <34 weeks		Preterm birth <32 weeks		Preterm birth <28 weeks		PPROM	
	Crude OR/RR (95%CI)	Adjusted OR/RR (95%CI)	Crude OR/RR (95%CI)	Adjusted OR/RR (95%CI)	Crude OR/RR (95%CI)	Adjusted OR/RR (95%CI)	Crude OR/RR (95%CI)	Adjusted OR/RR (95%CI)	Crude OR/RR (95%CI)	Adjusted OR/RR (95%CI)
Eze et al.	1.72 (0.37-7.91)	NR							1.27 (0.17-9.20)	NR
Zhao et al.	1.12 (0.99-1.27)	1.00 (0.90-1.20)							1.15 (1.05-1.26)	NR
Stout et al.	1.52 (1.34-1.72)	1.50 (1.30-1.80)	1.40 (1.12-1.76)	1.40 (1.00-1.80)					1.39 (1.09-1.78)	1.30 (1.00-1.70)
Chen et al.	1.46 (1.33-1.61)	1.32 (1.19-1.46)								
Girault et al.	1.44 (0.88-2.37)	2.90 (1.70-4.90)	1.78 (0.96-3.30)	NR					0.90 (0.40-2.04)	NR
Lai et al., Qidwai et al.	1.63 (1.27-2.10)	1.45 (1.08-1.96)	1.82 (1.27-2.59)	1.70 (1.12-2.58)	1.93 (1.26-2.94)	1.9 (1.17-3.20)	2.01 (1.18-3.41)	1.99 (1.05-3.75)	1.65 (0.98-2.80)	1.54 (0.82-2.89)
Blitz et al.	1.95 (1.51-2.53)	NR	2.44 (1.66-3.58)	NR	2.10 (1.28-3.44)	NR	2.48 (1.38-4.44)	NR		
Ciavattini et al.	2.33 (1.11-4.88)	NR							16.03 (2.10-122)	NR
Arisoy et al.	4.68 (1.88-11.6)	NR	4.24 (1.30-13.9)	NR	3.3 (0.8-13.4)	NR			6.73 (1.40-32.3)	NR
Egbe et al.	1.52 (0.53-4.42)	NR								

NR *not reported, no adjusted analysis was performed*
