# Supplementary figures and images for: The risk of preterm birth in women with uterine fibroids: A systematic review and meta-analysis
Source: PLoS One. 2022 Jun 2;17(6):e0269478. doi: 10.1371/journal.pone.0269478 (PMC9162311; doi:10.1371/journal.pone.0269478)

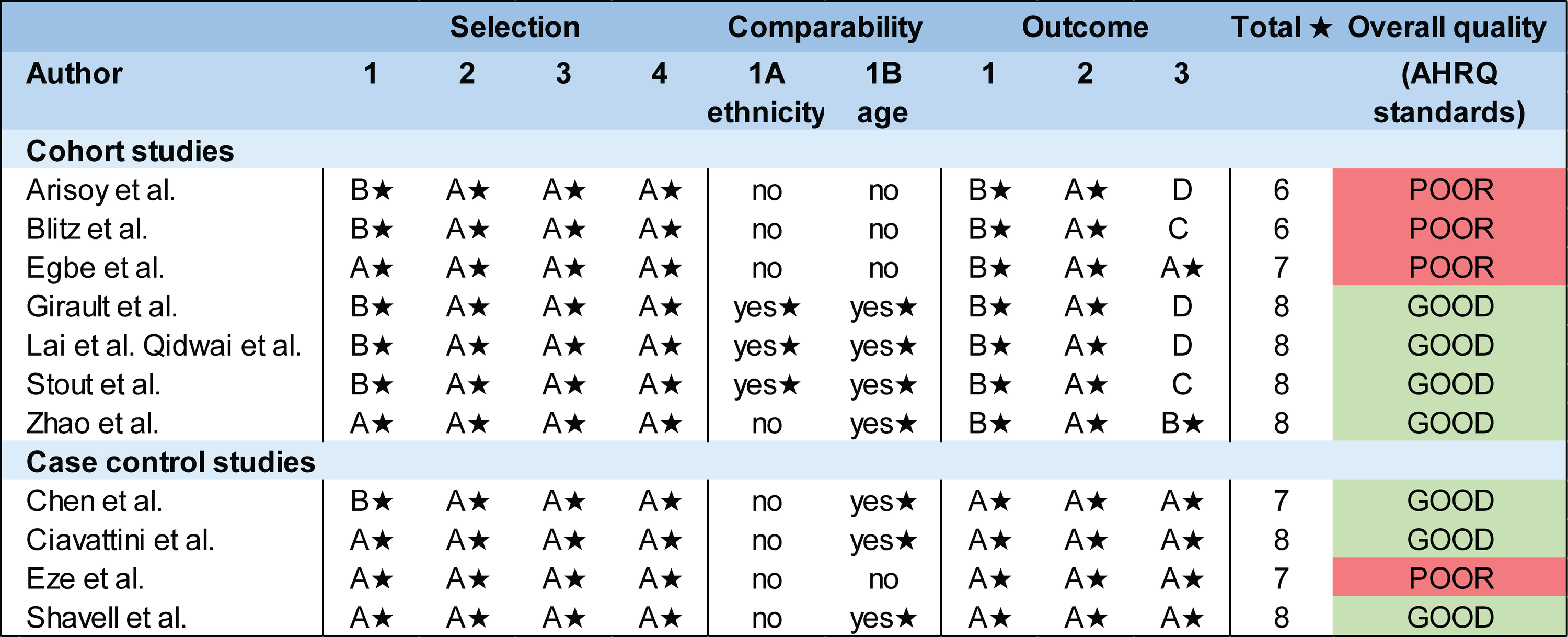

Supplement: S1 Fig — (TIF) [file pone.0269478.s007.tif]

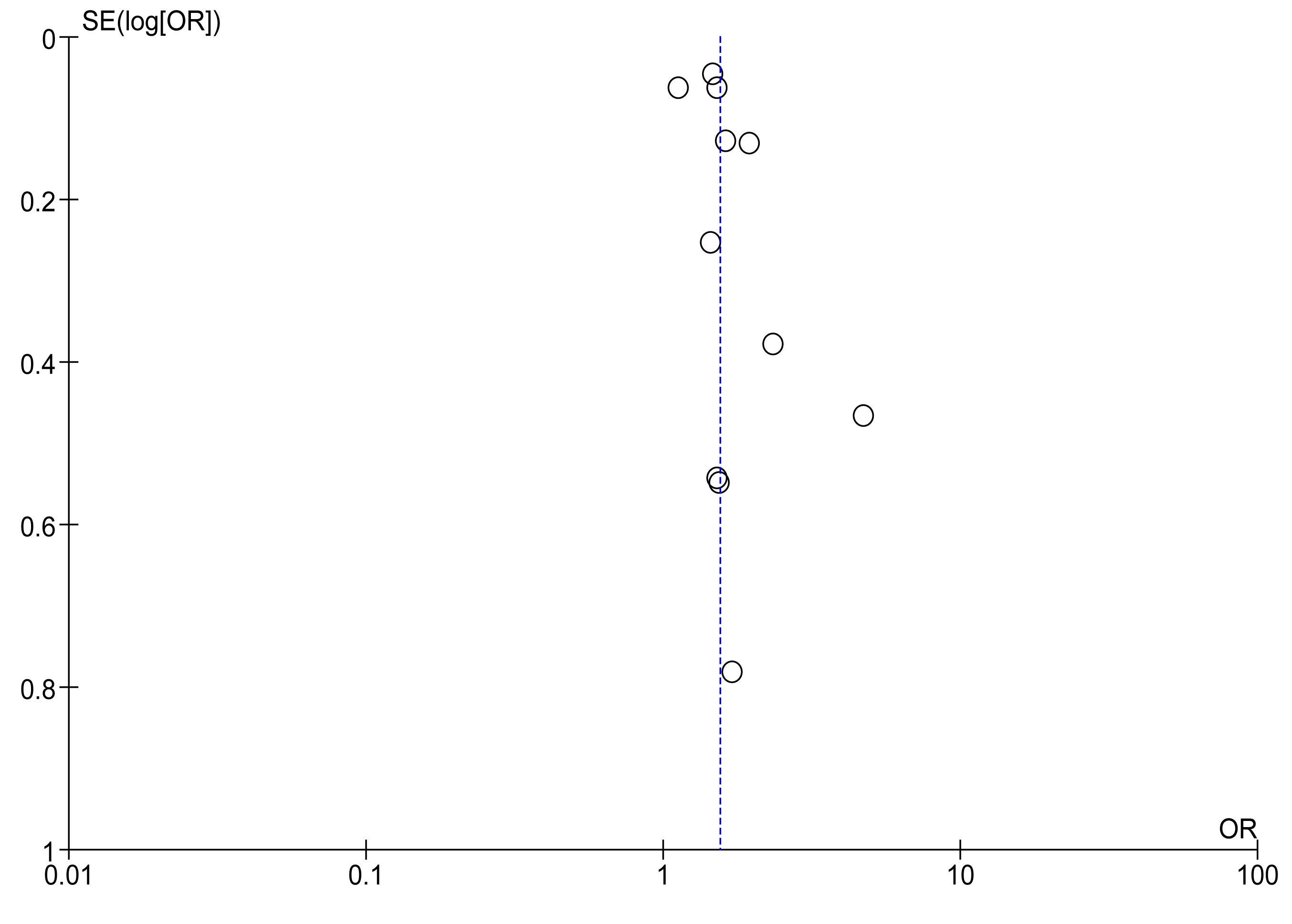

Supplement: S2 Fig — (TIF) [file pone.0269478.s008.tif]

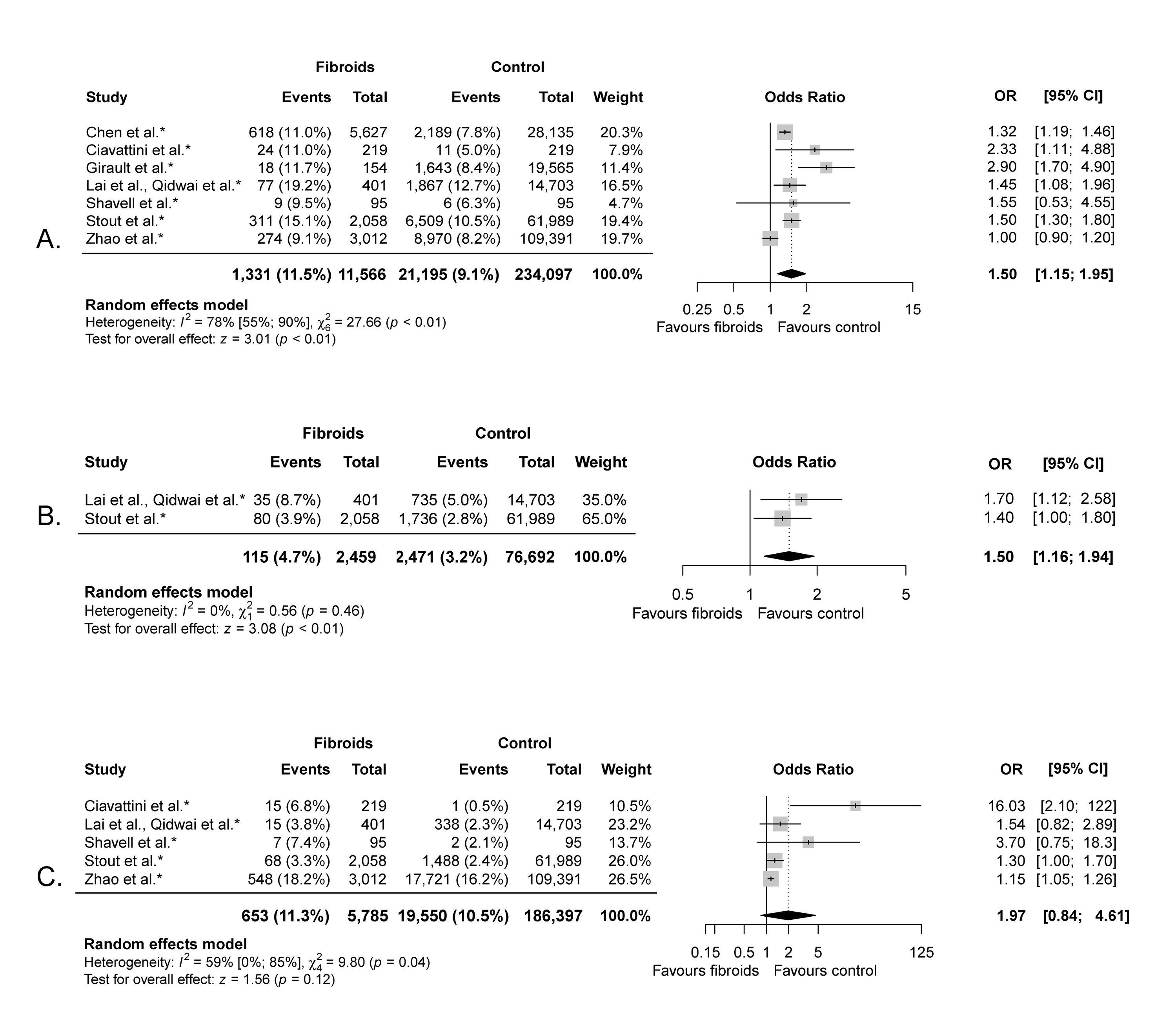

Supplement: S3 Fig — Sensitivity analyses of good quality studies for preterm birth (A) <37 and (B) <34 gestational weeks and (C) PPROM. * indicates studies that corrected for potential confounders. (TIF) [file pone.0269478.s009.tif]

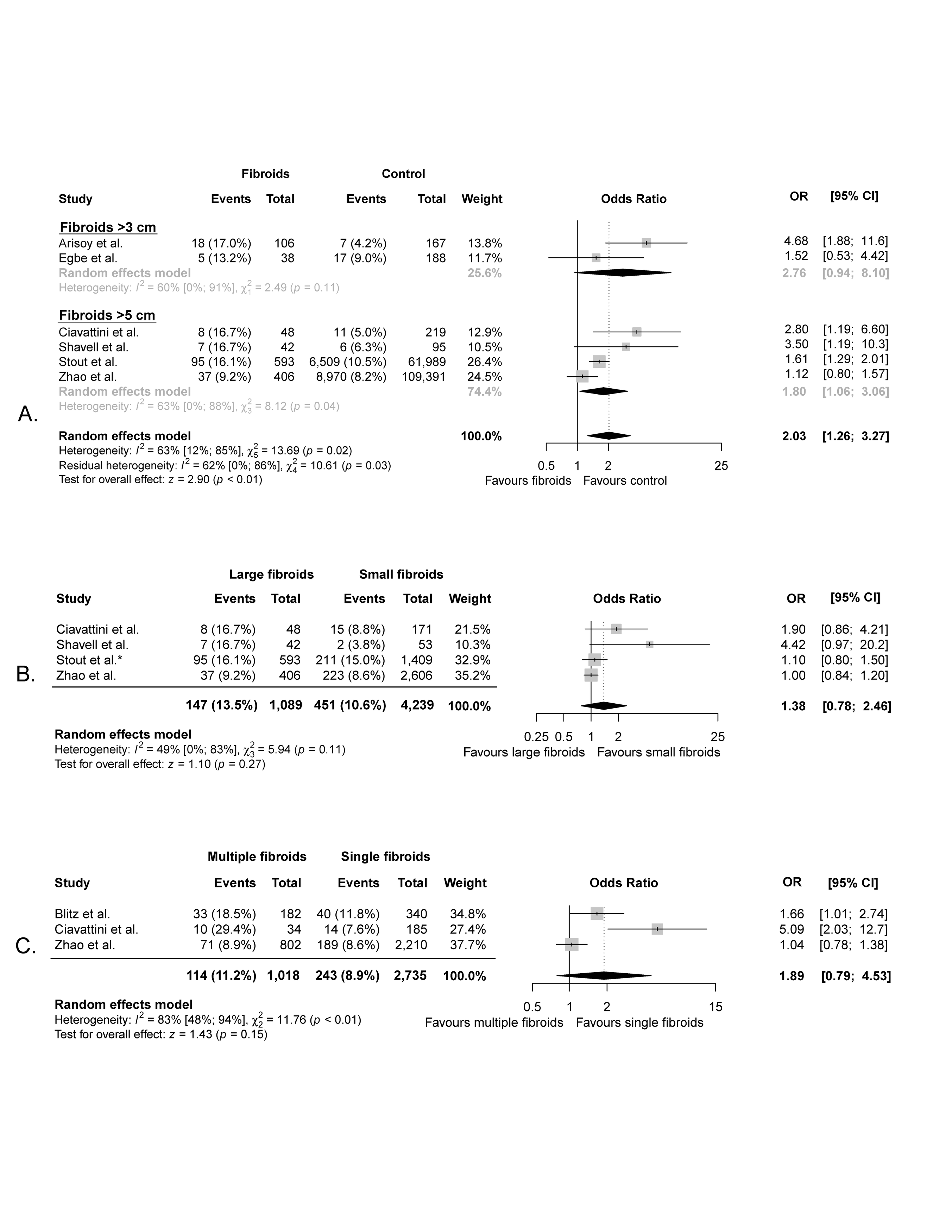

Supplement: S4 Fig — Meta-analysis of (A) fibroids >3 and >5 cm compared to women without fibroids; (B) large versus small fibroids; and (C) multiple versus single fibroids. * indicates studies that corrected for potential confounders. (TIF) [file pone.0269478.s010.tif]
